# Theabrownin from Dark Tea Attenuates Age-Related Cognitive Decline in Naturally Aged Mice by Modulating Gut Microbiota and Metabolites

**DOI:** 10.3390/foods15091587

**Published:** 2026-05-04

**Authors:** Mengjie Lei, Hang Xu, Xiaodi Jin, Xuemin Chen, Kezhuo Chen, Zixi Yang, Yanxia Xie, Dong Li, Mingzhang Ao, Yuanmin Zhu, Longjiang Yu

**Affiliations:** 1 Institute of Resource Biology and Biotechnology, Department of Biotechnology, College of Life Science and Technology, Huazhong University of Science and Technology, Wuhan 430074, China; d202280884@hust.edu.cn (M.L.); jinxiaodi1005@163.com (X.J.); chenxuemin@hust.edu.cn (X.C.); d202481124@hust.edu.cn (K.C.); zixi_yang@hust.edu.cn (Z.Y.); yanxia_xie@hust.edu.cn (Y.X.); d202080646@hust.edu.cn (D.L.); aomingzhang@hust.edu.cn (M.A.); 2 Key Laboratory of Molecular Biophysics, Ministry of Education, Wuhan 430074, China; 3 Sustainable Development Innovation Center, Huaxiang Innovation Research Institute, Linxiang District, Lincang 677000, China; 4 Hubei Key Laboratory of Purification and Application of Plant Anticancer Active Ingredients, School of Chemistry and Life Sciences, Hubei University of Education, Wuhan 430205, China; xuhang@hue.edu.cn

**Keywords:** theabrownin, dark tea, cognitive decline, gut microbiota, acetate, 3-hydroxybutyrate

## Abstract

Dietary factors play an important role in cognitive health during aging. Dark tea has shown potential cognitive benefits, but its key bioactive component and underlying mechanisms remain unclear. In a naturally aged C57BL/6J mouse model, instant dark tea (IDT) samples with different fermentation degrees were evaluated together with behavioral outcomes using composition–effect relationship analysis. This analysis identified theabrownin (TB) as the component most strongly associated with improved cognitive performance. Compared with aged controls, TB increased Y-maze spontaneous alternation from 51.91% to 71.59% and reduced escape latency on day 5 of the Morris water maze from 44.84 s to 26.59 s. In contrast, the corresponding TB-depleted fraction produced no comparable cognitive improvement. TB also alleviated hippocampal injury and neuroinflammation. Antibiotic treatment abolished the cognitive benefits of TB, whereas fecal microbiota transplantation partially restored them. Multi-omics analyses suggested that TB treatment was associated with gut microbiota remodeling and increased serum acetate and 3-hydroxybutyrate; both metabolites partially recapitulated these benefits. Together, these findings show that TB attenuates age-related cognitive decline in naturally aged mice and suggest that modulation of gut microbiota and metabolites may contribute to this effect, supporting its potential as a functional food ingredient for healthy brain aging.

## 1. Introduction

Age-related cognitive decline (ARCD) is an increasingly important public health issue in aging societies. ARCD not only substantially compromises the quality of life in older adults but also increases susceptibility to neurodegenerative disorders, particularly Alzheimer’s disease [1,2]. Epidemiological data indicate that approximately 20% of individuals over the age of 65 exhibit mild cognitive impairment, with an additional 10% affected by dementia [3,4]. Current pharmacological interventions, including cholinesterase inhibitors and N-methyl-D-aspartate (NMDA) receptor antagonists, provide limited symptomatic relief, fail to halt neurodegenerative progression, and are often associated with adverse effects [5,6]. Consequently, the development of effective and well-tolerated strategies to mitigate age-associated cognitive decline is urgently needed. Natural products, characterized by multitarget activities and favorable safety profiles, are increasingly recognized as promising sources of neuroprotective agents [7].

Among these natural products, dark tea, which undergoes microbial post-fermentation, has attracted increasing attention for its potential protective effects against cognitive decline [8]. In a 10-year longitudinal study of older Chinese adults, habitual tea consumption was associated with a 12% lower risk of cognitive decline [9]. Dark tea extracts also showed stronger anti-aging effects than extracts from other tea types in model organisms [10] and attenuated neuroinflammation in models of obesity-induced cognitive dysfunction [11,12], suggesting that compounds generated during post-fermentation may contribute to these effects. Theabrownin (TB), a characteristic high-molecular-weight pigment enriched in dark tea after microbial fermentation, is distinguished from low-molecular-weight tea constituents such as catechins, caffeine, and theanine by its complex polymeric structure [13,14]. Current evidence suggests that the biological effects of TB are primarily mediated by modulation of gut microbiota composition and function [15]. Given the important role of gut microbiota dysbiosis in age-related cognitive decline [16], TB may be a key bioactive constituent underlying dark tea’s protective effects. However, most existing studies have relied on whole tea or crude extracts, making it difficult to identify the specific constituents underlying these effects. In addition, mechanistic studies have mainly focused on obesity-related or experimentally induced aging models, and evidence under natural aging conditions remains limited [11,12,13]. Therefore, further studies under natural aging conditions are needed to identify the main bioactive constituents of dark tea and clarify the mechanisms underlying their protective effects.

In the present study, a liquid-state-fermented instant dark tea (IDT) preparation was employed to investigate the active constituents and mechanisms underlying the cognition-related effects of dark tea. This preparation provided a chemically reproducible experimental system for studying this complex fermented food. The compositional and structural characteristics of its major polymeric fraction have been characterized previously [17,18]. Naturally aged mice were used, with intervention initiated at 18 months of age and maintained for 6 months, to evaluate their long-term effects under conditions more closely reflecting the natural aging process. The ability of IDT to attenuate cognitive decline during natural aging was first examined, and composition–effect relationship analysis across IDT samples with different fermentation degrees was then combined with fraction-subtraction assays to identify the key active fraction. Antibiotic treatment, fecal microbiota transplantation (FMT), multi-omics profiling, and metabolite replenishment were further employed to investigate the potential mechanisms underlying its effects. Overall, this study may support the targeted development of cognition-related functional components from dark tea and their use in nutritional strategies for healthy cognitive aging.

## 2. Materials and Methods

### 2.1. Animals and Experimental Design

Eight-week-old male C57BL/6J mice of specific pathogen-free (SPF) grade were supplied by Henan Skbex Biotechnology Co., Ltd. (Anyang, China) and housed in a controlled environment at 20–22 °C, 45 ± 5% relative humidity, and a 12 h light/dark cycle, with ad libitum access to standard chow and water. Mice were housed in groups of five per cage in ventilated cages with autoclaved bedding. Mice from the same batch were randomly assigned to the indicated experimental groups at the beginning of the study using random numbers generated in Microsoft Excel. They were then maintained under standard laboratory conditions until 18 months of age. The corresponding interventions were initiated at 18 months and continued for 6 months. Young control mice were purchased at 8 weeks of age at the beginning of each intervention and maintained in parallel for 6 months. At the end of the intervention period, behavioral tests were performed, followed by euthanasia by CO_2_ inhalation. Blood, feces, hippocampi, and whole brains were collected. No prespecified inclusion or exclusion criteria were applied, and no animals, experimental units, or data points were excluded from the final analyses. Investigators were not blinded during treatment administration or behavioral testing because the tea extracts differed visibly in color; however, outcomes were quantified using automated tracking or objective instrumental methods.

The study consisted of five experimental parts, and all test substances were provided in drinking water for 6 months. Unless otherwise stated, each group included 10 mice, consistent with previous studies using naturally aged mice [19]. In the first part, mice were used to evaluate the effects of instant dark tea on aging-related alterations and were assigned to the young control (CON), aging model (MOD), and IDT groups. The IDT group received IDT at 4 mg/mL, whereas the CON and MOD groups received purified water. In the second part, mice were used to identify candidate bioactive components of IDT and were assigned to the CON, MOD, IDT-1, IDT-2, IDT-3, and IDT-4 groups. The treatment groups received the corresponding tea extracts at 4 mg/mL, whereas the CON and MOD groups received purified water. Candidate bioactive components were identified based on behavioral outcomes combined with Spearman correlation analysis. In the third part, mice were used to verify the candidate bioactive component and were assigned to the CON, MOD, IDT-4, theabrownin (TB), and theabrownin-depleted fraction (DTBT) groups. The IDT-4 group received IDT-4 at 4 mg/mL, whereas the TB and DTBT groups received their respective preparations at the same concentration (2 mg/mL). This concentration was selected to enable direct comparison between the paired fractions and was close to the estimated theabrownin-equivalent level in the 4 mg/mL IDT-4 treatment.

In the fourth part, mice were used to determine whether the effects of TB were dependent on the gut microbiota and were assigned to the TB, TB + AB, TB + FMT, MOD FMT, and TB FMT groups. The TB group received TB for 6 months. The TB + AB group received TB for 6 months and an antibiotic cocktail (ABX) during the final 2 months. The TB + FMT group received TB for 6 months, together with ABX during the first 2 months, followed by fecal microbiota transplantation from TB donors during the subsequent 4 months. The MOD FMT and TB FMT groups received ABX during the first 2 months, followed by FMT from MOD or TB donors, respectively, during the subsequent 4 months, without additional TB treatment. In the fifth part, mice were used to assess the contribution of gut microbiota-related metabolites and were assigned to the MOD, 3-hydroxybutyrate (3-HB), and acetate (AA) groups. The 3-HB and AA groups received 3-HB (3 mg/mL) or acetate (67.5 mM), respectively, whereas the MOD group received purified water. Detailed procedures for ABX treatment, donor feces preparation, and FMT are provided in Section 2.3. To reduce animal use, some groups were shared across related experiments. Specifically, the CON, MOD, and IDT-4 cohorts were shared between the first and second experimental parts, and the TB cohort was shared between the third and fourth experimental parts.

### 2.2. Preparation and Chemical Composition of Intervention Materials

Instant dark tea and tea powders with different fermentation durations (IDT-1 to IDT-4) were prepared according to our previously established method [17]. Briefly, sun-dried green tea inoculated with *P. camelliae* Z09 (Lincang Dianhai Ganquan Tea Factory, Lincang, China) was fermented for 0, 24, 36, or 48 h to obtain IDT-1, IDT-2, IDT-3, and IDT-4, respectively. In the present study, IDT-4 was used as the intervention material referred to as IDT in Section 2.1. Theabrownin was purified as previously described [17]. The theabrownin-depleted fraction was prepared from the non-TB fractions obtained during TB purification. Soluble sugars, tea polyphenols, gallic acid, caffeine, catechins, theaflavins, thearubigins, theabrownins, free amino acids, and total flavonoids were quantified using established colorimetric, spectrophotometric, and HPLC-based methods as previously described [17,20].

### 2.3. Specific Animal Procedures

Antibiotic treatment was performed according to a modified protocol [21]. Mice received 100 μL of a broad-spectrum antibiotic cocktail by intragastric gavage once daily for 1 week, followed by administration on alternate days during the corresponding treatment period described in Section 2.1. The antibiotic mixture used for microbiota depletion consisted of ampicillin, neomycin sulfate, and gentamicin at 1 mg/mL each, together with streptomycin sulfate at 0.5 mg/mL. All antibiotics were obtained from Shanghai Macklin Biochemical Co., Ltd. (Shanghai, China).

Fecal microbiota transplantation was performed according to a previously established protocol [22]. Fecal samples from the designated donor mice were collected aseptically on the day of transplantation and homogenized in pre-cooled sterile PBS (Servicebio, Wuhan, China) to 200 mg/mL. The mixture was thoroughly vortexed, centrifuged at 1500 rpm for 5 minutes to pellet coarse debris, and the supernatant containing gut microbiota was retained. Recipient mice received 200 μL of the freshly prepared fecal microbiota suspension by oral gavage daily for the initial 5 days, followed by weekly administration during the subsequent transplantation period to promote microbiota engraftment.

### 2.4. Behavioral Tests

The Y-maze test was used to assess spatial working memory [23]. Each mouse was placed at the center of a three-arm maze—with the arms arranged at 120° angles—and allowed to explore the apparatus freely for 8 minutes. The maze was wiped with 10% ethanol (Macklin, Shanghai, China) between trials to remove olfactory cues. Spontaneous alternation was calculated as: number of alternations/(total arm entries − 2) × 100.

Spatial learning and memory were assessed in a 120-cm-diameter circular pool using the Morris water maze (MWM) test [24]. During the acquisition phase, mice were trained four times daily for 5 consecutive days, with a maximum search time of 60 s to locate the hidden platform. On day 6, a probe trial was conducted with the platform removed, during which the time spent in the target quadrant and swimming trajectories were recorded. Swimming trajectories were analyzed using ToxTrac 2.93 software [25].

### 2.5. Histological and Immunofluorescence Analysis

Brain tissues embedded in paraffin were cut into 5 μm coronal sections. Neuronal morphology was assessed by Nissl staining using a commercial kit (Servicebio, Wuhan, China) and imaged with an upright optical microscope (Olympus, Tokyo, Japan) [21]. For immunofluorescence analysis, sections underwent antigen retrieval and blocking, followed by overnight incubation at 4 °C with anti-PSD95 and anti-Iba1 antibodies (1:200; Servicebio, Wuhan, China). After incubation with fluorophore-conjugated secondary antibodies (Servicebio, Wuhan, China) and DAPI (Servicebio, Wuhan, China) counterstaining, fluorescence images were recorded with a digital pathology scanner (3DHISTECH, Budapest, Hungary) [26].

### 2.6. Biochemical and Targeted Metabolite Analysis

Hippocampal tissues were homogenized in PBS, subjected to freeze–thaw cycles, and centrifuged at 7300 rpm for 5 minutes at 4 °C to collect the supernatants, which were used to measure TNF-α, IL-6, and IL-1β using ELISA kits (Jiangsu Meimian Industrial, Yancheng, China).

Serum was separated by centrifugation of whole blood at 2000× *g* for 10 min at 4 °C. Serum 3-hydroxybutyrate was measured using a commercial assay kit (Beyotime Biotechnology, Shanghai, China) in accordance with the manufacturer’s protocol. In addition, serum short-chain fatty acids (SCFAs) were analyzed by gas chromatography–mass spectrometry (GC–MS) following a previously reported method [27]. Briefly, acidified serum was extracted with anhydrous ether (Sinopharm, Shanghai, China), dried over Na_2_SO_4_ (Solarbio, Beijing, China), derivatized with BSTFA (Aladdin, Shanghai, China), and then analyzed on an Agilent GC–MS system (Agilent Technologies, Santa Clara, CA, USA).

### 2.7. Omics Analyses

Fecal 16S rDNA sequencing was performed to characterize gut microbial composition. Fecal genomic DNA was isolated using a commercial extraction kit from Ark Bioscience (Guangzhou, China). The bacterial 16S rRNA gene V3–V4 region was amplified, followed by high-throughput sequencing on the Illumina NovaSeq 6000 platform by Magigene Biotechnology (Guangzhou, China). Operational taxonomic units were generated at a 97% sequence similarity threshold with UCLUST and taxonomically assigned using the Greengenes database [21].

Serum untargeted metabolomic profiling was conducted using a UHPLC–Orbitrap MS platform from Thermo Fisher Scientific (Waltham, MA, USA), operated in both positive and negative electrospray ionization modes. Serum proteins were precipitated with cold methanol/acetonitrile (Macklin, Shanghai, China) containing isotope-labeled internal standards. Data were processed using the XCMS package in R, and metabolites were identified based on MS1/MS2 spectral matching [21]. Further methodological details are provided in the Supplementary Materials and Methods.

Hippocampal transcriptomic analysis was performed by RNA sequencing. Total hippocampal RNA was isolated with TRIzol reagent from Meiji Biotechnology (Guangzhou, China), followed by library sequencing on the Illumina NovaSeq 6000 platform. Differential gene expression analysis was conducted using DESeq2 with thresholds of FDR ≤ 0.05 and |log_2_(FoldChange)| ≥ 1 [26].

### 2.8. Quantitative Real-Time PCR

Total RNA from hippocampal tissue was reverse-transcribed into cDNA and subjected to quantitative real-time PCR (qRT-PCR) using SYBR Green chemistry (Nanjing Vazyme Biotech, Nanjing, China) following a previously published protocol [24]. β-Actin served as the reference gene, and relative transcript levels were determined by the 2^−ΔΔCt^ method. The primer pairs used in this study are provided in Table S2.

### 2.9. Statistical Analysis

All data are presented as the mean ± standard error of the mean (SEM). Statistical evaluation was carried out using IBM SPSS Statistics 27.0.1 (IBM, Armonk, NY, USA). Differences between the two groups were assessed with an unpaired two-tailed Student’s t-test. In contrast, comparisons among multiple groups were analyzed by one-way analysis of variance (ANOVA) followed by Tukey’s post hoc multiple-comparison test. *p* < 0.05 was considered statistically significant.

## 3. Results

### 3.1. IDT Intervention Improves Cognitive Performance in Naturally Aged Mice

**Figure 1:**
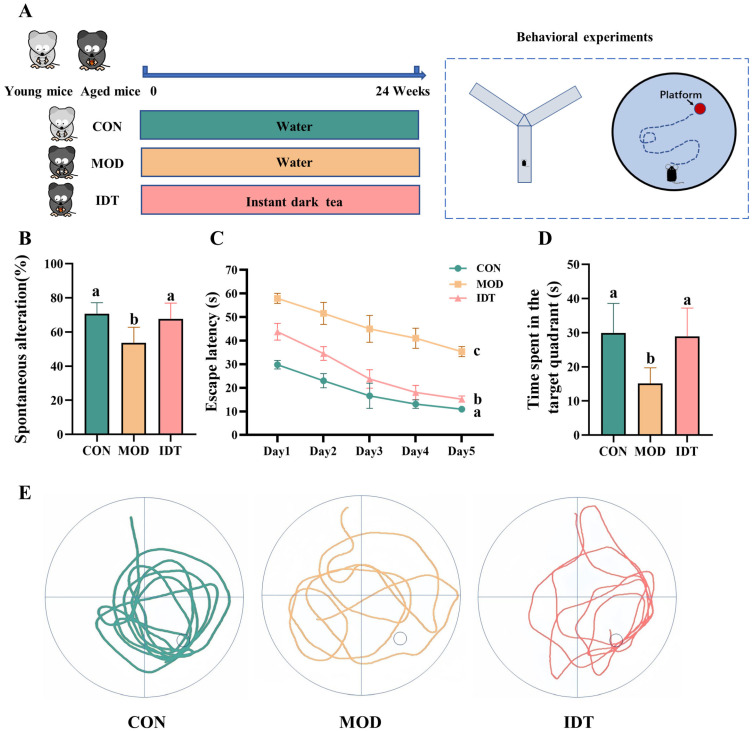
Effects of instant dark tea on cognitive performance in naturally aged mice. (**A**) Experimental design. (**B**) Y-maze spontaneous alternation rate. (**C**) Escape latency in the MWM test. (**D**) Time spent in the target quadrant. (**E**) Representative swimming trajectories. Data are presented as mean ± SEM (*n* = 8 per group). Different letters indicate significant differences (*p* < 0.05).

To evaluate spatial working memory and long-term spatial learning, mice underwent the Y-maze and Morris water maze, respectively [23,24]. Y-maze testing showed an age-associated decline in spontaneous alternation, from 70.75 ± 6.06% in CON mice to 53.64 ± 8.57% in MOD mice (*p* < 0.05), consistent with impaired spatial working memory. IDT treatment increased the alternation rate to 67.63 ± 8.74% (*p* < 0.05 vs. MOD; Figure 1B).

In the MWM test, the MOD group showed a significantly longer escape latency than the CON group during the acquisition phase. On day 5, the escape latency was 35.34 ± 2.01 s in the MOD group and 10.91 ± 0.89 s in the CON group (*p* < 0.05). IDT treatment significantly shortened the latency to 15.19 ± 1.22 s (*p* < 0.05; Figure 1C), representing a 20.15 s reduction relative to the MOD group. On day 6, target-quadrant residence time was lower in the MOD group than in the CON group (15.13 ± 4.31 s vs. 29.88 ± 8.15 s, *p* < 0.05), whereas IDT treatment significantly increased target-quadrant residence time (Figure 1D,E). These results suggest that IDT intervention improved cognitive performance in naturally aged mice, particularly in spatial working memory and long-term spatial learning.

### 3.2. Theabrownin Was Identified as a Major Contributor to the Cognitive Effects of IDT

**Figure 2:**
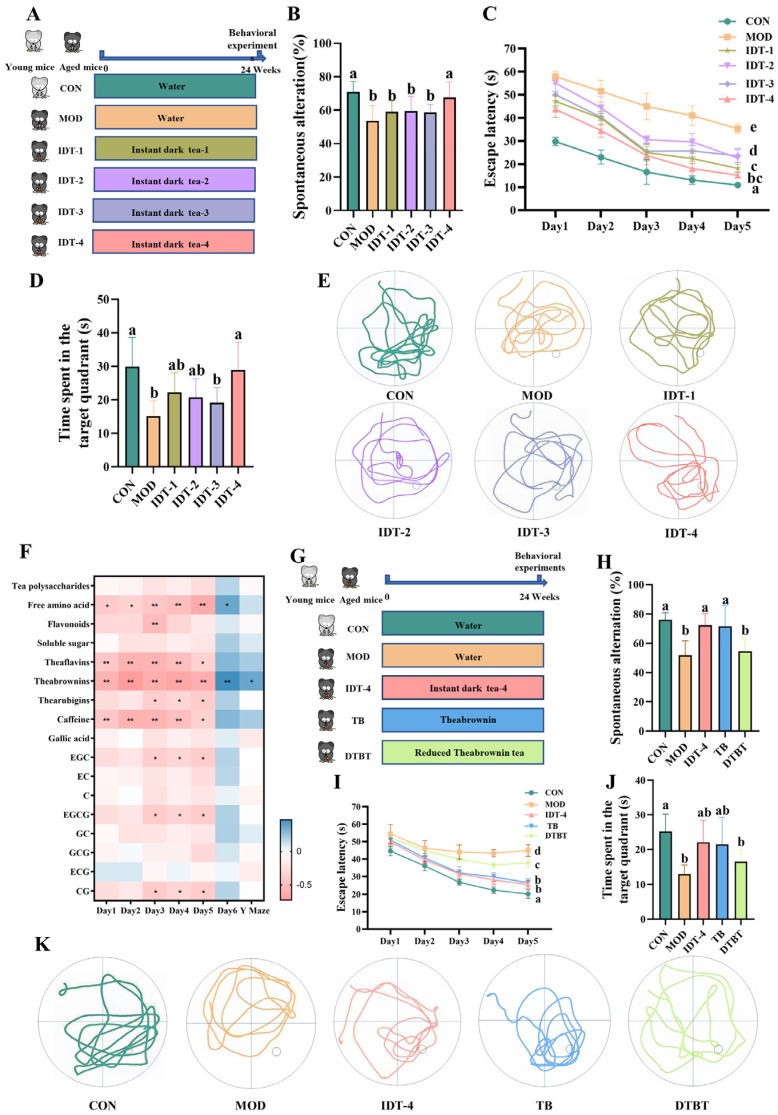
Identification of TB as the key bioactive constituent in fermented tea that protects against age-related cognitive decline. (**A**–**E**) Cognitive effects of IDT samples with different fermentation durations: (**A**) experimental design; (**B**) Y-maze spontaneous alternation rate; (**C**) escape latency in the MWM test; (**D**) time spent in the target quadrant; (**E**) representative swimming trajectories. (**F**) Spearman’s correlation heatmap between behavioral indices and major tea constituents. (**G**–**K**) Comparative effects of TB and DTBT: (**G**) experimental design. (**H**) Y-maze spontaneous alternation rate. (**I**) Escape latency in the MWM test. (**J**) Time spent in the target quadrant. (**K**) Representative swimming trajectories. Data are presented as mean ± SEM (*n* = 8 per group). Different letters indicate significant differences (*p* < 0.05). * *p* < 0.05 and ** *p* < 0.01.

To identify the constituents associated with the cognitive effects of instant dark tea, naturally aged mice were administered tea extracts prepared at different fermentation levels (IDT-1 to IDT-4; Figure 2A). The major chemical components of IDT-1 to IDT-4 are summarized in Table S1. In the Y-maze test, IDT-1, IDT-2, and IDT-3 did not significantly improve spontaneous alternation compared with the MOD group, whereas IDT-4 significantly increased the alternation rate from 53.64 ± 8.57% in the MOD group to 67.63 ± 8.74% (*p* < 0.05; Figure 2B). Similarly, in the MWM test, IDT-4 significantly reduced the day-5 escape latency from 35.34 ± 2.01 s in the MOD group to 15.19 ± 1.22 s (*p* < 0.05; Figure 2C), 20.15 s shorter than the MOD group, and increased the time spent in the target quadrant during the probe trial from 15.13 ± 4.31 s to 28.88 ± 7.83 s (*p* < 0.05; Figure 2D). These results indicate that the cognitive effects were most pronounced in the highly fermented preparation IDT-4.

Spearman rank correlation analysis between chemical composition and behavioral indices showed a positive association between theabrownin content and cognitive performance (Figure 2F). Consistent with this result, IDT-4 contained the highest level of theabrownin among the tested preparations (Table S1). Based on these findings, theabrownin was selected for further validation.

To validate the contribution of TB, the cognitive effects of IDT-4, TB, and the TB-depleted fraction were compared. In the Y-maze test, TB increased the spontaneous alternation rate from 51.91 ± 9.19% in the MOD group to 71.59 ± 13.26% (*p* < 0.05; Figure 2H), close to that observed in the IDT-4 group (72.39 ± 7.38%). In contrast, DTBT did not improve spontaneous alternation (54.47 ± 8.21%). In the MWM test, TB treatment significantly reduced escape latency to 26.59 ± 2.00 s, representing an 18.25 s decrease relative to the MOD group, and increased target-quadrant residence time to 21.50 ± 7.30 s. In contrast, DTBT did not improve either escape latency (37.78 ± 2.52 s) or target-quadrant residence time (16.50 ± 3.16 s) (Figure 2I,J). Together, these findings support theabrownin as a major contributor to the cognitive effects of highly fermented IDT.

### 3.3. Theabrownin Attenuates Hippocampal Neuronal Damage and Neuroinflammation in Aged Mice

**Figure 3:**
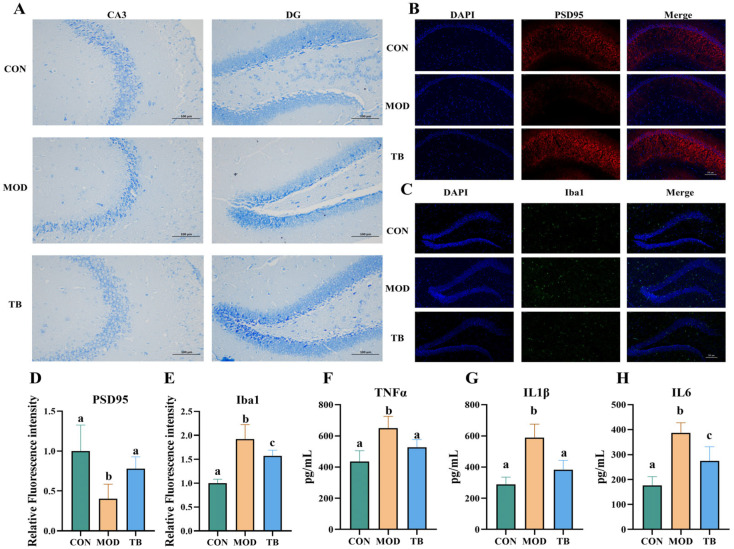
Theabrownin attenuates neuronal damage and neuroinflammation in aged mice. (**A**) Representative images of Nissl staining. (**B**,**D**) PSD95 immunofluorescence and quantification. (**C**,**E**) Iba1 immunofluorescence and quantification. (**F**–**H**) Brain inflammatory cytokine levels. Data are presented as mean ± SEM (*n* = 6 per group). Different letters indicate significant differences (*p* < 0.05).

To characterize hippocampal tissue-level changes associated with TB treatment, hippocampal neuronal morphology, synaptic marker expression, and neuroinflammatory status were examined. Histological assessment via Nissl staining revealed that neurons in the MOD group exhibited reduced density, disordered architecture, and shrunken morphology, features consistent with hippocampal neuronal injury. In contrast, hippocampal sections from TB-treated mice displayed a more compact and orderly neuronal architecture, comparable to that observed in the CON group (Figure 3A). The expression of the postsynaptic marker PSD95 was evaluated by immunofluorescence. Quantitative analysis of fluorescence intensity demonstrated a significant reduction in PSD95 expression in the MOD group relative to the CON group (*p* < 0.05), indicating reduced PSD95 immunoreactivity. TB administration significantly increased PSD95 fluorescence intensity (*p* < 0.05) (Figure 3B,D), suggesting higher postsynaptic marker expression after TB treatment.

Neuroinflammatory status was subsequently assessed by characterizing microglial activation and pro-inflammatory cytokine levels. Iba1 immunofluorescence analysis revealed markedly elevated microglial activation in the MOD group, as evidenced by increased relative Iba1 fluorescence intensity compared with the CON group (*p* < 0.05). TB treatment significantly attenuated this activation, resulting in a substantial reduction in Iba1 signal relative to the MOD group (*p* < 0.05) (Figure 3C,E). Consistently, TNF-α, IL-1β, and IL-6 levels were also increased in the MOD group (*p* < 0.05), whereas TB treatment reduced the levels of all three cytokines (*p* < 0.05) (Figure 3F–H). Collectively, these findings indicate that theabrownin treatment ameliorated hippocampal neuronal morphological abnormalities, helped preserve synaptic integrity, and attenuated neuroinflammatory responses in aged mice.

### 3.4. The Gut Microbiota Is Required for the Cognitive Benefits of Theabrownin

**Figure 4:**
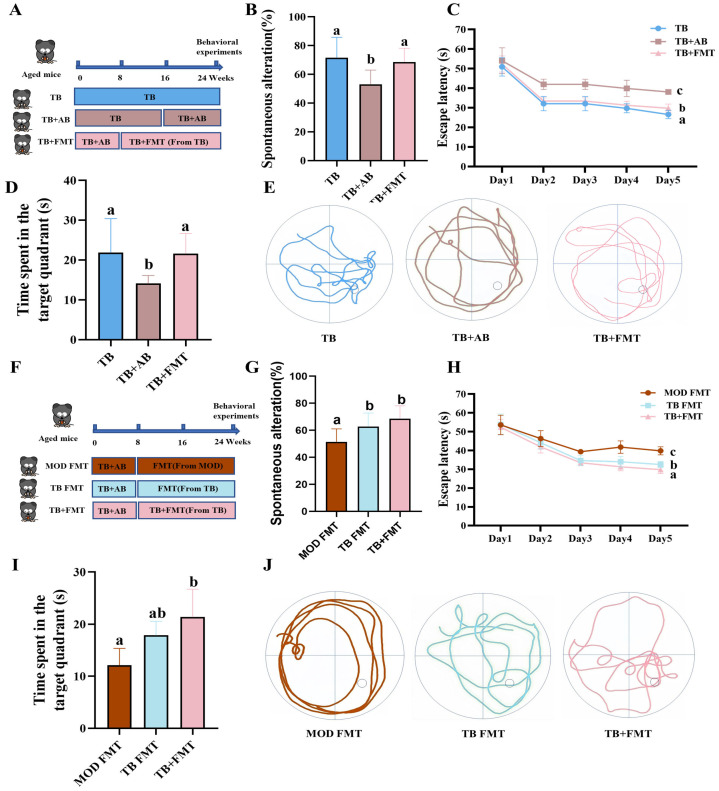
The cognitive-improving effects of theabrownin depend on the gut microbiota. (**A**–**E**) Antibiotic-induced microbiota depletion experiment: (**A**) experimental design; (**B**) Y-maze spontaneous alternation rate; (**C**) escape latency in the MWM test; (**D**) time spent in the target quadrant; (**E**) representative swimming trajectories. (**F**–**J**) Fecal microbiota transplantation experiment: (**F**) experimental design; (**G**) Y-maze spontaneous alternation rate; (**H**) escape latency in the MWM test; (**I**) time spent in the target quadrant; (**J**) representative swimming trajectories. Data are presented as mean ± SEM (*n* = 8 per group). Different letters indicate significant differences (*p* < 0.05).

To determine whether the gut microbiota contributed to the cognitive benefits of TB, TB-treated mice were subjected to antibiotic-mediated microbiota depletion followed by fecal microbiota transplantation as indicated (Figure 4A). In the Y-maze, the TB + AB group showed a lower spontaneous alternation rate than the TB group (53.13 ± 9.19% vs. 71.59 ± 13.26%, *p* < 0.05; Figure 4B). In the MWM test, antibiotic treatment similarly worsened performance, as reflected by a longer escape latency (38.13 ± 1.01 s in TB + AB vs. 26.59 ± 2.00 s in TB, *p* < 0.05) and a reduced target-quadrant residence time (14.13 ± 1.90 s vs. 21.88 ± 7.98 s, *p* < 0.05). In contrast, FMT partially restored these deficits. Relative to the TB + AB group, the TB + FMT group showed a spontaneous alternation rate of 68.63 ± 8.92%, a reduced escape latency of 29.81 ± 1.90 s, and an increased target-quadrant residence time of 21.63 ± 4.74 s compared with the TB + AB group (*p* < 0.05; Figure 4C,D). These observations suggest that microbiota depletion attenuated the behavioral improvements associated with TB treatment, whereas FMT partially reversed these changes.

To further determine whether the gut microbiota confers cognitive protection independently or acts synergistically with TB, additional FMT experiments were performed (Figure 4F). In the Y-maze, recipient mice receiving microbiota from TB-treated donors (TB FMT) showed a significantly higher spontaneous alternation rate than those receiving microbiota from model donors (MOD FMT) (62.82 ± 9.37% vs. 51.47 ± 8.96%, *p* < 0.05; Figure 4G). Consistently, the TB FMT group showed a shorter escape latency than the MOD FMT group (32.53 ± 1.48 s vs. 39.78 ± 2.02 s, *p* < 0.05) and greater target-quadrant residence time (17.88 ± 2.52 s vs. 12.13 ± 3.02 s, *p* < 0.05; Figure 4H,I). Moreover, the TB + FMT group showed better Morris water maze performance than both the TB FMT and MOD FMT groups, as reflected by a shorter escape latency and a longer target-quadrant residence time (*p* < 0.05; Figure 4H,I). Collectively, these findings suggest that the gut microbiota contributes to TB-associated improvements in spatial learning and memory, and that the presence of TB, together with a reconstituted gut microbiota, is associated with greater behavioral improvement in aged mice.

### 3.5. Theabrownin Remodels Gut Microbiota Composition

**Figure 5:**
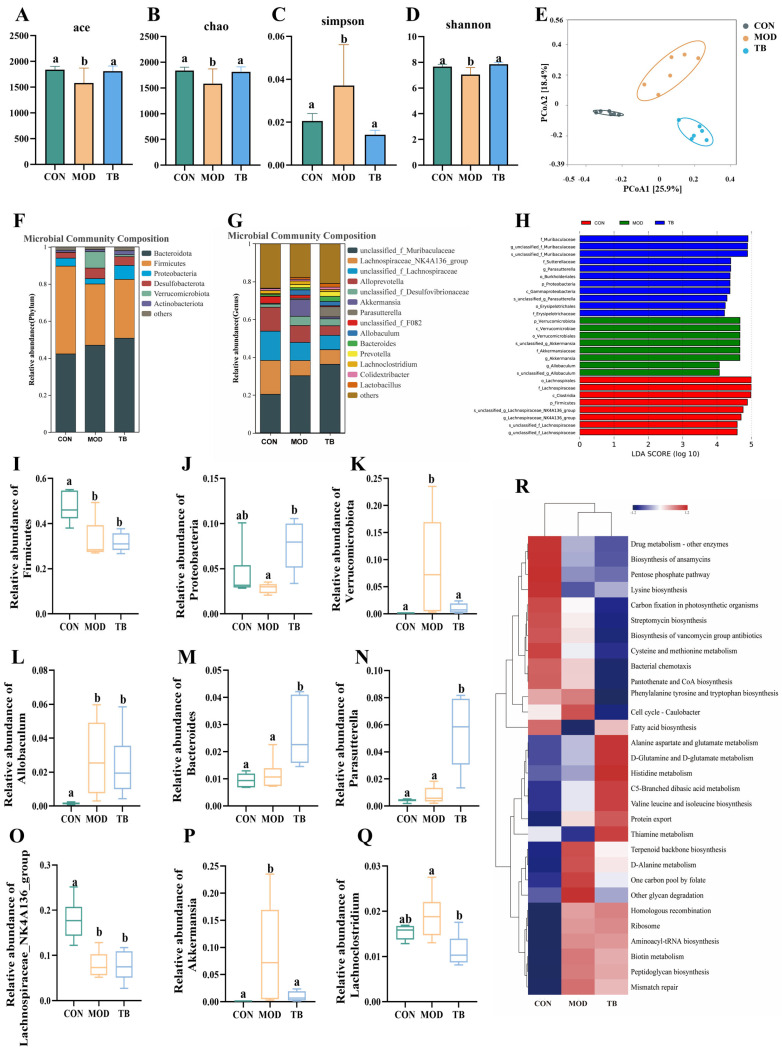
16S rDNA sequencing analysis and functional prediction of the gut microbiota. (**A**–**D**) α-diversity indices. (**E**) β-diversity analysis. (**F**,**G**) Relative abundance of gut microbiota at the phylum and genus levels. (**H**) LEfSe-identified taxonomic biomarkers. (**I**–**K**) Top 10 differential taxa at the phylum level. (**L**–**Q**) Top 10 differential taxa at the genus level. (**R**) Predicted KEGG metabolic pathways. Data are presented as mean ± SEM (*n* = 6 per group). Different letters indicate significant differences (*p* < 0.05).

16S rDNA sequencing analysis showed that TB intervention was associated with higher gut microbiota diversity indices. Specifically, the TB group had higher Chao1, ACE, and Shannon indices and a lower Simpson index than the MOD group (*p* < 0.05; Figure 5A–D). PCoA revealed distinct clustering of the TB and MOD groups (Figure 5E). Taxonomic profiling further indicated that TB treatment induced marked compositional shifts at both the phylum and genus levels. At the phylum level, the relative abundances of Verrucomicrobiota and Proteobacteria were higher in the TB group than in the MOD group, and the relative abundance of Proteobacteria was also elevated compared with that in the CON group (*p* < 0.05; Figure 5I–K). At the genus level, TB treatment decreased the relative proportions of *Lachnoclostridium* and *Akkermansia*, while increasing those of *Bacteroides* and *Parasutterella* (*p* < 0.05; Figure 5L–Q). Overall, TB intervention was associated with marked alterations in gut microbiota composition in aged mice.

To further identify microbial biomarkers and explore their potential functional relevance, LEfSe and PICRUSt2 analyses were performed. LEfSe analysis showed that the MOD group was characterized by enrichment of *Lachnospirales*, *Clostridia*, and *Akkermansiaceae*, whereas TB treatment was associated with enrichment of *Muribaculaceae*, *Sutterellaceae*, *Erysipelotrichaceae*, and *Burkholderiales* (Figure 5H). PICRUSt2 analysis further suggested that these taxonomic changes were accompanied by shifts in predicted microbial functions. Predicted pathways associated with fatty acid biosynthesis and the metabolism of alanine, aspartate, glutamate, and branched-chain amino acids were enriched after TB intervention relative to the MOD group (Figure 5R). Together, these findings suggest that TB remodeled both gut microbiota composition and predicted microbial metabolic functions in aged mice.

### 3.6. Serum Metabolites 3-Hydroxybutyrate and Acetate Are Associated with the Cognitive Benefits of Theabrownin

**Figure 6:**
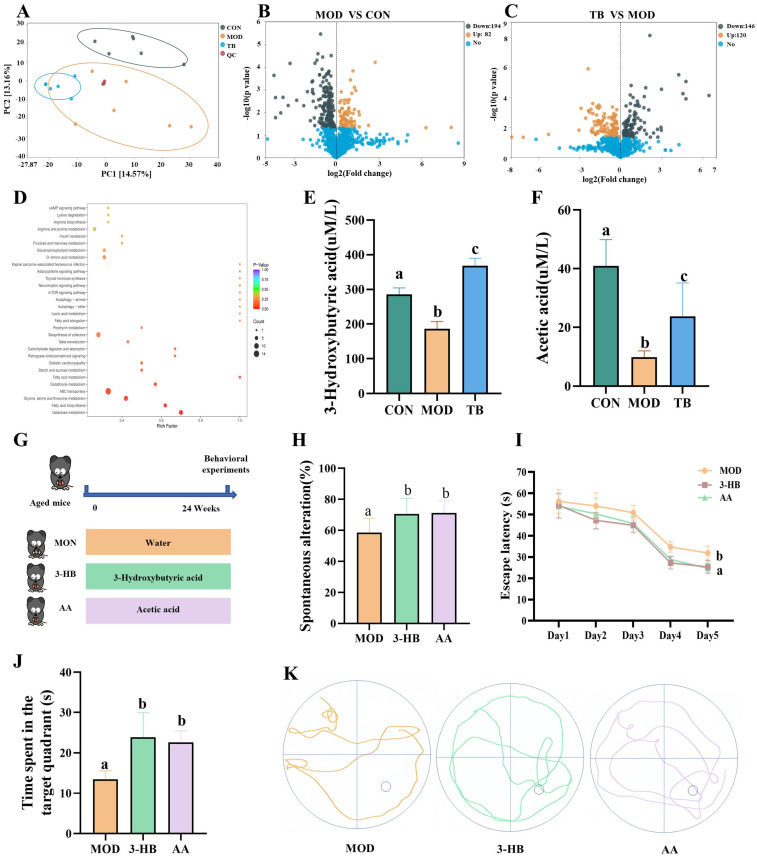
Serum metabolites associated with theabrownin treatment partially reproduce cognitive improvement. (**A**) Principal component analysis score scatter plot of all serum samples. (**B**,**C**) Volcano plots showing differential serum metabolites in the MOD versus CON and TB versus MOD comparisons. (**D**) KEGG pathway enrichment analysis of differential metabolites between the TB and MOD groups. (**E**,**F**) Serum levels of 3-hydroxybutyrate and acetate. (**G**) Experimental design for metabolite supplementation. (**H**) Y-maze spontaneous alternation rate. (**I**) Escape latency in the MWM test. (**J**) Time spent in the target quadrant. (**K**) Representative swimming trajectories. Data are presented as mean ± SEM; *n* = 6 per group for metabolomics analysis (**A**–**F**) and *n* = 8 per group for behavioral tests (**H**–**J**). Different letters indicate significant differences (*p* < 0.05).

To characterize serum metabolic alterations associated with TB treatment, untargeted metabolomics analysis was performed. Principal component analysis (PCA) showed a distinct separation between the TB and MOD groups, reflecting different serum metabolomic profiles following TB treatment (Figure 6A). Relative to the MOD group, 266 metabolites were significantly changed in the TB group, including 120 that increased and 146 that decreased (Figure 6C). KEGG enrichment analysis further indicated that these altered metabolites were predominantly associated with amino acid metabolism and fatty acid biosynthesis (Figure 6D). These pathway-level changes were consistent with the predicted microbial functional shifts identified by PICRUSt2 analysis.

Spearman rank correlations were calculated to assess associations between gut microbial composition and host metabolic phenotypes (Figure S1). The results showed that several differentially abundant genera, including *Gordonibacter*, *Ruminococcus*, *Bacteroides*, *Ileibacterium*, and *Atopostipes*, were positively correlated with multiple circulating metabolites, such as 3-hydroxybutyrate and hippuric acid (*p* < 0.05). In contrast, metabolites including xipamide and trans-4-hydroxycinnamic acid sulfate exhibited negative correlations with most of these bacterial taxa. Based on these association profiles, metabolites closely correlated with TB-enriched genera were further selected for targeted quantification. Among these metabolites, 3-HB was prioritized for further validation based on its strong correlation with multiple TB-enriched taxa and its established ability to cross the blood–brain barrier. Subsequent targeted detection confirmed a significant increase in serum 3-HB after TB treatment (Figure 6E). In parallel, *Bacteroides*, a genus commonly associated with short-chain fatty acid production, was enriched in the TB group. Targeted analysis confirmed that serum acetate levels were significantly elevated in the TB group (Figure 6F). Together, these results identified 3-HB and acetate as candidate microbiota-associated metabolites potentially linked to TB-induced metabolic remodeling.

To assess whether these metabolites contributed to the behavioral improvement, aged mice were supplemented with either 3-HB or acetate and then subjected to behavioral testing. In the Y-maze, both 3-HB and acetate significantly increased the spontaneous alternation rate to 70.52 ± 9.49% and 71.28 ± 7.22%, respectively, compared with 58.65 ± 8.66% in the MOD group (*p* < 0.05; Figure 6H). In the Morris water maze, both treatments significantly shortened escape latency (25.38 ± 2.74 s for 3-HB and 24.69 ± 2.24 s for acetate) relative to the MOD group (31.88 ± 2.98 s), with reductions of 6.50 s and 7.19 s, respectively (*p* < 0.05; Figure 6I). During the probe trial, 3-HB and acetate supplementation significantly increased target-quadrant residence time compared with the MOD group (13.44 ± 1.98 s), corresponding to increases of 10.42 and 9.15 s, respectively (*p* < 0.05; Figure 6J). Overall, these results suggest that 3-HB and acetate partially reproduced the cognitive improvement observed after TB treatment, supporting their roles as candidate downstream metabolites.

### 3.7. Theabrownin Reshapes the Hippocampal Transcriptomic Profile and Modulates Neurotransmission-Related Signaling

**Figure 7:**
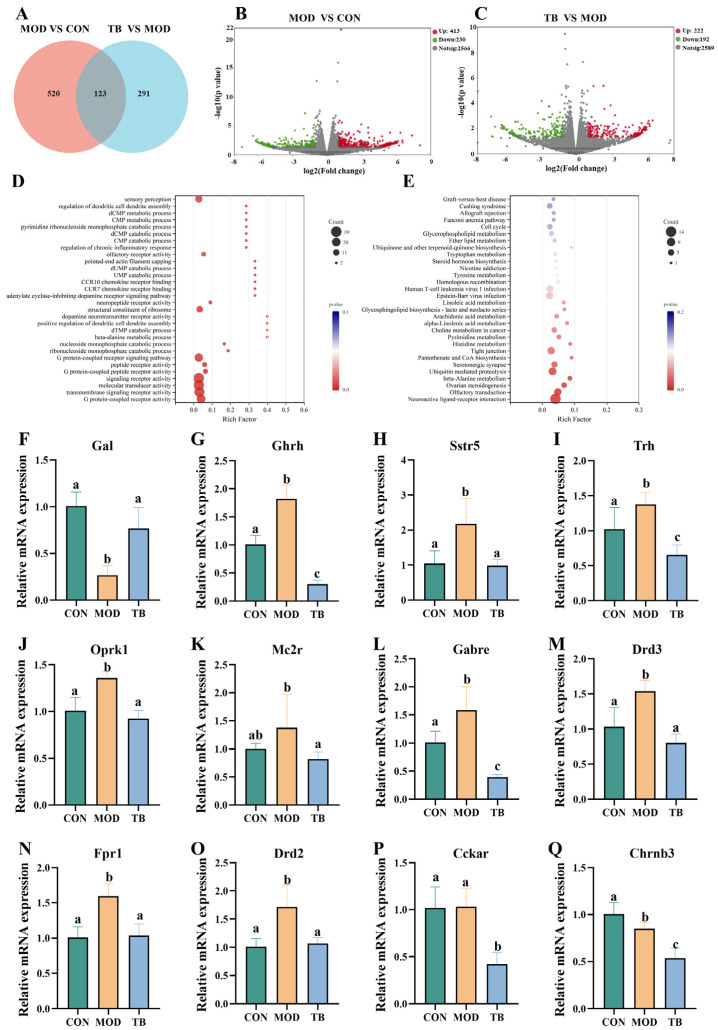
Transcriptomic analysis of the hippocampus. (**A**) Venn diagram of differentially expressed genes (DEGs). (**B**,**C**) Volcano plots showing DEGs in the MOD versus CON and TB versus MOD comparisons. (**D**,**E**) GO and KEGG enrichment analyses of DEGs between the TB and MOD groups. (**F**–**Q**) Hippocampal mRNA expression levels of selected DEGs. Data are presented as mean ± SEM; *n* = 3 per group for transcriptomic analysis (**A**–**E**) and *n* = 6 per group for qPCR validation (**F**–**Q**). Different letters indicate significant differences (*p* < 0.05).

To characterize the impact of TB intervention on hippocampal transcriptional changes in aged mice, transcriptomic sequencing was performed on hippocampal tissues from the CON, MOD, and TB groups. Venn diagram analysis identified 123 overlapping differentially expressed genes (DEGs) shared between aging-associated changes (MOD vs. CON) and TB-associated changes (TB vs. MOD) (Figure 7A). Relative to the MOD group, TB treatment significantly altered the expression of 414 genes, including 222 upregulated and 192 downregulated genes, indicating broad transcriptional remodeling in the hippocampus (Figure 7C). To explore the biological relevance of these transcriptional changes, functional enrichment analyses were performed. Gene Ontology analysis showed that the DEGs were predominantly enriched in signal transduction-related categories, including G protein-coupled receptor activity, dopamine neurotransmitter receptor activity, and transmembrane signaling receptor activity (Figure 7D). KEGG analysis further identified neuroactive ligand–receptor interaction as a prominently enriched pathway (Figure 7E). Overall, marked changes in hippocampal signaling-related gene expression were associated with TB treatment.

To validate the RNA-seq results, representative DEGs within the neuroactive ligand–receptor interaction pathway were further quantified by qPCR. Aging was associated with altered expression of *Gal* and multiple receptor-related genes, including *Drd2*, *Drd3*, *Fpr1*, *Oprk1*, and *Sstr5* (*p* < 0.05). TB intervention partially normalized several of these aging-associated changes, increasing *Gal* expression and reducing *Drd2*, *Drd3*, *Fpr1*, and *Oprk1* expression (*p* < 0.05) (Figure 7F–Q). These genes are involved in synaptic transmission (including *Drd2*, *Drd3*, and *Gal*) and neuroinflammatory processes (including *Fpr1* and *Oprk1*) [28,29]. Together, these transcriptomic and qPCR results indicate that TB treatment was associated with hippocampal gene expression changes within the neuroactive ligand–receptor interaction pathway.

## 4. Discussion

Age-related cognitive decline is a growing public health concern worldwide and has been linked to synaptic dysfunction, chronic neuroinflammation, and gut microbiota dysregulation [16,30]. However, effective interventions capable of delaying or counteracting this process remain limited. Nutritional intervention has emerged as a practical and sustainable approach for mitigating cognitive decline in daily life [31,32]. Epidemiological evidence links regular tea consumption to a lower risk of cognitive impairment and dementia in older adults [33]. Nevertheless, most mechanistic studies to date have focused on catechin-rich teas or extracts and have mainly relied on models of acute injury or accelerated aging [34]. Unlike unfermented teas, dark tea undergoes microbial post-fermentation, which alters its chemical composition and may confer distinct biological activities [35]. Accordingly, the cognitive benefits of fermented dark tea during natural aging, as well as the material basis underlying these effects, remain to be clarified.

In contrast to conventional studies based on acute injury or accelerated-aging models, the present work employed a 6-month dietary intervention in naturally aged mice. This prolonged intervention more closely reflects the physiological course of cognitive aging and thus provides a more relevant context for evaluating long-term dietary strategies [36]. Under these conditions, behavioral assessments using the Y-maze and Morris water maze showed that long-term IDT supplementation ameliorated age-associated deficits in spatial working memory and learning. Nevertheless, identifying the specific bioactive basis within the chemically complex system of dark tea remains challenging. To address this issue, relationships between chemical composition and cognitive phenotypes were examined using instant dark tea samples across a fermentation gradient. Notably, the extensively fermented fraction (IDT-4) significantly improved cognitive performance, whereas the less fermented fractions (IDT-1 to IDT-3) produced only limited effects, with several indices showing no significant difference from the model group. This fermentation-dependent pattern suggests that constituents accumulating during microbial post-fermentation may be related to the observed behavioral effects. Subsequent composition-efficacy correlation analysis identified theabrownin, a characteristic oxidative polymer enriched in IDT-4, as the main candidate constituent. Comparative intervention experiments were then performed to further assess this relationship in vivo. Supplementation with the TB fraction reproduced the behavioral improvements observed with IDT-4, whereas the DTBT failed to produce comparable cognitive benefits. Taken together, these findings suggest that TB may be an important contributor to the cognitive benefits of fermented IDT.

After identifying TB as a principal bioactive constituent, its effects on the hippocampal microenvironment were further examined. Cognitive function depends strongly on synaptic integrity, whereas chronic neuroinflammation is an important contributor to age-related neurodegeneration [37,38]. In the present study, TB intervention alleviated pathological alterations in the aging hippocampus by reducing neuronal damage, restoring PSD95 expression, and suppressing excessive microglial activation. During natural aging, overactivated microglia promote the release of pro-inflammatory cytokines, which can damage neurons and disrupt synaptic integrity [38]. By suppressing microglial activation and reducing inflammatory markers, TB alleviated the neuroinflammatory burden in the aged hippocampus. This protective effect on synapse-associated proteins accords with recent evidence indicating that Qingzhuan dark tea-derived theabrownin can suppress glial activation and increase PSD95 expression under high-fat diet-induced neural injury conditions [11]. The present findings extend those observations by showing that TB can also alleviate neuroinflammation and preserve synaptic integrity during natural physiological aging, rather than only under conditions of acute metabolic insult. Overall, suppression of inflammatory responses and preservation of synaptic homeostasis in the aging hippocampus may, at least in part, underlie the cognitive benefits of TB.

The present findings further support the involvement of the gut microbiota in TB-associated cognitive improvement. This was assessed through antibiotic-induced microbiota depletion and fecal microbiota transplantation. Behavioral results indicated that ABX weakened the beneficial effects of TB, whereas FMT from TB-treated donors partially transferred these effects. These results suggest that gut microbiota-mediated processes may contribute, at least in part, to the cognitive benefits associated with TB. However, the partial transfer by FMT indicates that other contributory mechanisms cannot be excluded. This interpretation is consistent with previous studies showing that theabrownin derived from Pu-erh tea ameliorates hypercholesterolemia, through gut microbiota remodeling and downstream metabolic regulation [15]. On this basis, the present findings further support an important mediating role of the gut microbiota in the systemic effects of theabrownin. It is also noteworthy that combined TB supplementation and FMT produced greater cognitive improvement than FMT alone. This may indicate that TB may be associated with alterations in gut microbiota composition and may also serve as a fermentable substrate that generates secondary metabolites through microbial activity, thereby contributing to neuroprotection. However, this possibility remains to be further verified. At the same time, the highly complex polymeric structure of TB and its diverse metabolic pathways make comprehensive characterization of its in vivo metabolic fate challenging, and this remains an important area for future investigation [39].

16S rDNA sequencing further showed that TB partially reshaped the age-related gut microbial profile. TB reversed the age-related reduction in α-diversity, which is commonly regarded as an indicator of microbial community stability [40]. At the genus-level composition, TB increased *Bacteroides* and *Parasutterella* abundance. Bacteroides is a major degrader of dietary macromolecules and an important producer of short-chain fatty acids [41]. In contrast, *Parasutterella* is considered a core gut microbial taxon associated with host metabolic homeostasis, particularly with respect to bile acid maintenance and cholesterol metabolism [42]. In contrast, TB significantly reduced the abundance of *Lachnoclostridium*, a taxon frequently overrepresented in dysbiosis and associated with systemic inflammation and cognitive impairment [43]. TB also normalized the age-related overabundance of *Akkermansia*. Although *Akkermansia* is generally considered beneficial as a mucin-degrading bacterium, its excessive expansion under conditions of impaired intestinal barrier function may aggravate mucus erosion and metabolic disturbance in aged hosts [44,45]. These discrepant findings may be related to differences in host age, intestinal barrier status, and pathological context. Therefore, the reduction in age-related Akkermansia enrichment following TB treatment may indicate alleviation of gut microbial dysbiosis rather than supporting a detrimental role for this genus. In addition, LEfSe analysis identified *Muribaculaceae* and *Sutterellaceae* as the core differential taxa associated with the microbial shift after TB treatment. The enrichment of *Sutterellaceae* is taxonomically consistent with the expansion of its subordinate genus, *Parasutterella*. Likewise, the expansion of *Muribaculaceae*, a keystone family specialized in the utilization of complex carbohydrates, may contribute to improved microbial energy metabolism during aging [40]. PICRUSt2 functional prediction further suggested that these taxonomic changes were associated with enhanced pathways related to fatty acid biosynthesis and amino acid metabolism. As PICRUSt2 provides inferred rather than direct functional evidence, these pathway-level changes should be regarded as TB-associated microbial functional signatures requiring further validation.

Metabolomic analysis further provided a circulating-metabolite perspective on the relationship between TB-associated microbial alterations and host metabolic responses. Systemic metabolic changes, particularly those involving amino acid and fatty acid metabolism, partially matched the PICRUSt2-predicted amino acid and fatty acid metabolism pathways, supporting pathway-level consistency between microbial functional predictions and serum metabolomic profiles. Integrated Spearman correlation analysis showed that TB-enriched microbial taxa were positively associated with elevated circulating levels of multiple metabolites. Among these metabolites, 3-HB was strongly correlated with key bacterial taxa and has been reported to cross the blood–brain barrier, where it may act in the brain as an alternative energy substrate and an epigenetically active metabolite [46,47]. These reported properties support the biological plausibility of 3-HB as a candidate downstream metabolite, although its precise contribution to TB effects requires further validation. In parallel, TB increased serum acetate levels, consistent with the enrichment of the short-chain fatty acid-producing genus Bacteroides. Given that acetate has been reported to attenuate inflammatory responses through GPR43-mediated regulation of NLRP3 inflammasome activity [48], this change is also consistent with the reduced hippocampal inflammatory response observed after TB treatment. Given their established roles in gut–brain communication, 3-HB and acetate were further examined as candidate downstream mediators. Exogenous supplementation with either metabolite partially reproduced the cognitive improvements observed with TB treatment, supporting their potential involvement in TB-associated cognitive protection. Overall, TB intervention was associated with attenuated age-related cognitive decline, gut microbiota remodeling, and increased circulating 3-HB and acetate levels. These microbial and metabolic alterations provide a peripheral basis for TB-associated cognitive protection and support further investigation of the corresponding central molecular responses.

To explore the central molecular responses accompanying these peripheral metabolic alterations, hippocampal transcriptomics was performed. The analysis identified the neuroactive ligand–receptor interaction pathway as one of the major pathways affected by TB intervention. Natural aging was associated with increased expression of *Fpr1* and *Oprk1*, receptors related to microglial activation and neuroinflammation. In contrast, TB treatment significantly downregulated these genes, in parallel with the observed suppression of microglial activation and cerebral inflammatory factors [28,29]. This anti-inflammatory transcriptional profile is consistent with the elevated serum acetate level observed after TB treatment. It may reflect a potential association between peripheral metabolic changes and central inflammatory responses. In addition, aging altered dopaminergic signaling, as indicated by the upregulation of *Drd2* and *Drd3* transcripts, which have been associated with working memory decline [49,50]. TB normalized the transcriptional levels of these receptors, which may have contributed to synaptic homeostasis, and concurrently upregulated the expression of *Gal*, which encodes the neuroprotective neuropeptide galanin [51]. At the same time, the TB-associated increase in serum 3-HB, together with the reported epigenetic activity of 3-HB [46,47], may be related to the observed transcriptional changes and warrants further investigation. Overall, these transcriptomic findings suggest that TB treatment was associated with central molecular responses related to neuroinflammation, neurotransmission, and synaptic regulation, providing a molecular perspective on the hippocampal protection and cognitive improvement observed in this study.

These findings extend the biological relevance of fermented dark tea beyond conventional low-molecular-weight polyphenols and suggest that microbiota-associated metabolites may contribute to the gut–brain effects of TB. Nevertheless, several limitations should be considered, including the largely correlative nature of the multi-omics data, the indirect causal evidence provided by ABX, FMT, and metabolite supplementation experiments, the use of only male mice, relatively modest sample sizes, the absence of formal power calculation and dose–response analysis, limited validation of omics findings, potential behavioral confounders, lack of a pharmacological positive control, and untested batch-to-batch reproducibility of TB. In addition, the dose used here is more relevant to a TB-enriched functional ingredient than to a single serving of conventionally brewed tea, and its human bioavailability and translational relevance require further evaluation. Future studies using larger sex-balanced cohorts, dose–response designs, multiple TB batches, positive controls, and targeted functional validation are needed to further clarify the metabolic fate, microbial biotransformation, and cognitive relevance of TB.

## 5. Conclusions

In conclusion, this study supports theabrownin as a key bioactive component of instant dark tea associated with the attenuation of age-related cognitive decline in naturally aged mice. Theabrownin reshaped the age-associated gut ecosystem, enriched beneficial microbial taxa such as *Bacteroides*, and was accompanied by increased levels of acetate and 3-hydroxybutyrate. Supplementation with either metabolite alone partially reproduced the cognitive benefits of theabrownin, supporting their potential functional relevance. These systemic alterations were accompanied by improved hippocampal synaptic integrity, reduced neuroinflammation, and normalization of neuroactive ligand–receptor signaling. Together, these findings provide preclinical evidence supporting the potential of theabrownin as a fermented tea-derived functional ingredient for cognitive health during aging. Further studies are needed to define its dose–response relationship, bioavailability, safety, and efficacy in humans.

## Data Availability

All data supporting the findings of this study are provided in the article and Supplementary Materials; additional requests may be addressed to the corresponding authors.

## Supplementary Materials

The following supporting information can be downloaded at https://www.mdpi.com/article/10.3390/foods15091587/s1, Materials and methods; Table S1: Major chemical components of IDT-1–IDT-4; Table S2: The primer sequences of qRT-PCR; Figure S1: Hierarchical clustering heatmap of correlations between gut microbiota and serum metabolites.

## Abbreviations

The following abbreviations are used in this manuscript:

IDT	instant dark tea
ARCD	age-related cognitive decline
NMDA	N-methyl-D-aspartate
BBB	blood–brain barrier
SCFAs	short-chain fatty acids
FMT	fecal microbiota transplantation
SPF	specific pathogen-free
MOD	model group
CON	control group
DTBT	theabrownin-depleted fraction
ABX	antibiotic cocktail
3-HB	3-hydroxybutyrate
AA	acetate
PBS	phosphate-buffered saline
MWM	Morris water maze
PSD95	postsynaptic density protein 95
Iba1	ionized calcium-binding adapter molecule 1
ELISA	enzyme-linked immunosorbent assay
TNF-α	tumor necrosis factor-α
IL-1β	interleukin-1β
IL-6	interleukin-6
SEM	standard error of the mean
PCoA	principal coordinate analysis
PCA	principal component analysis
DEGs	differentially expressed genes
