# Supplementary material for: Theabrownin from Dark Tea Attenuates Age-Related Cognitive Decline in Naturally Aged Mice by Modulating Gut Microbiota and Metabolites
Source: Foods. 2026 May 4;15(9):1587. doi: 10.3390/foods15091587 (PMC13163565; doi:10.3390/foods15091587)
Supplement: Supplementary file 1 [file foods-15-01587-s001.zip › foods-4264729-supplementary.pdf]

Theabrownin from Dark Tea Attenuates Age-Related Cognitive Decline in Naturally Aged Mice by Modulating Gut Microbiota and Metabolites

Mengjie Lei [1,2,3,†], Hang Xu [4,†], Xiaodi Jin [1,2,3], Xuemin Chen [1,2,3], Kezhuo Chen [1,2,3], Zixi Yang [1,2,3], Yanxia Xie [1,2,3], Dong Li [1,2,3], Mingzhang Ao [1,2], Yuanmin Zhu [1,2,*] and Longjiang Yu [1,2,*]

[1] Institute of Resource Biology and Biotechnology, Department of Biotechnology, College of Life Science and Technology, Huazhong University of Science and Technology, Wuhan 430074, China; d202280884@hust.edu.cn (M.L.); Jinxiaodi1005@163.com (X.J.); chenxuemin@hust.edu.cn (X.C.); d202481124@hust.edu.cn (K.C.); zixi_yang@hust.edu.cn (Z.Y.); yanxia_xie@hust.edu.cn (Y.X.); d202080646@hust.edu.cn (D.L.); aomingzhang@hust.edu.cn (M.A.)

[2] Key Laboratory of Molecular Biophysics, Ministry of Education, Wuhan 430074, China

[3] Huaxiang Innovation Research Institute, Linxiang District, Lincang (Sustainable Development Innovation Center, Linxiang District, Lincang), Lincang, Yunnan 677000, China

[4] Hubei Key Laboratory of Purification and Application of Plant Anticancer Active Ingredients, School of Chemistry and Life Sciences, Hubei University of Education, Wuhan 430205, China; xuhang@hue.edu.cn (H.X.)

* Correspondence: zhuyuanmin@hust.edu.cn (Y.Z.); yulongjiang@hust.edu.cn (L.Y.); Tel: +86-27-87792265

† These authors contributed equally to this work.

Materials and methods：Serum Untargeted Metabolomics Analysis

Table S1. Major chemical components of IDT1–IDT4.

Table S2. The primer sequences of RT-PCR.

Figure. S1. Hierarchical clustering heatmap of correlations between gut microbiota and serum metabolites.

Materials and methods

Serum Untargeted Metabolomics Analysis

Serum untargeted metabolomics analysis was performed using a Vanquish UHPLC system coupled to an Orbitrap Exploris 120 mass spectrometer (Thermo Fisher Scientific, Waltham, MA, USA) operated in both positive and negative electrospray ionization modes. Serum proteins were precipitated with cold methanol/acetonitrile containing isotope-labeled internal standards, followed by vortexing (30 s), ultrasonication, incubation at −40 °C for 1 h, and centrifugation for 15 min; the resulting supernatants were subjected to LC–MS analysis. Chromatographic separation was achieved on a Waters ACQUITY UPLC BEH Amide column using water and acetonitrile as the mobile phases, with a sample tray temperature of 4 °C and an injection volume of 2 μL. The mass source parameters were set as follows: sheath gas flow rate, 50 Arb; auxiliary gas flow rate, 15 Arb; capillary temperature, 320 °C; and spray voltage, 3.8 kV in positive mode and −3.4 kV in negative mode. Pooled quality control (QC) samples were prepared by mixing equal aliquots of supernatants from all samples and analyzed together with the study samples to monitor analytical stability. Raw data were converted to mzXML format using ProteoWizard and processed using an in-house R pipeline based on XCMS for peak detection, extraction, alignment, and integration. Features with poor reproducibility based on relative standard deviation filtering or with more than 50% missing values were removed; the remaining missing values were imputed using half of the minimum detected value, and peak intensities were normalized using isotope-labeled internal standards. Metabolites were putatively annotated by MS/MS spectral matching against a self-built secondary mass spectral database.

Tables

Table S1: Major chemical components of IDT1-IDT4

Constituent (mg/g)	IDT-1	IDT-2	IDT-3	IDT-4
Tea polyphenols	336.45±2.37^{a}	342.14±3.90^{a}	314.22±3.90^{b}	277.50±1.79^{c}
Free amino acid	48.14±0.48^{a}	33.74±0.31^{b}	27.16±0.23^{c}	27.67±0.37^{c}
Flavonoids	22.03±2.24^{a}	21.95±1.72^{a}	27.69±5.46^{a}	14.61±1.27^{b}
Soluble sugar	14.82±0.87^{a}	21.84±1.78^{c}	18.26±0.08^{b}	16.68±0.20^{ab}
Theaflavins	1.65±0.08^{a}	2.28±0.18^{b}	2.64±0.31^{b}	2.37±0.21^{b}
Theabrownins	30.04±1.03^{a}	108.40±11.88^{b}	191.25±35.45^{c}	394.04±53.09^{d}
Thearubigins	90.60±4.50	82.42±14.72	78.70±20.66	72.64±11.57
Caffeine	85.64±2.61^{a}	99.93±2.74^{b}	109.21±1.84^{c}	107.67±2.87^{c}
Gallic acid	52.17±1.64^{a}	3.25±0.27^{b}	0.42±0.05^{c}	0.00±0.00^{c}
EGC	25.24±0.93^{a}	48.93±1.18^{b}	7.27±0.32^{c}	0.00±0.00^{d}
EC	30.27±1.01^{a}	42.91±1.42^{b}	24.84±4.13^{c}	15.03±0.18^{d}
EGCG	51.88±1.29^{a}	18.74±4.16^{b}	4.81±0.42^{c}	3.36±0.15^{c}
ECG	52.19±1.24^{a}	27.13±2.36^{b}	10.16±1.55^{c}	5.37±0.82^{d}
GC	12.12±0.63^{a}	25.10±1.46^{b}	18.31±0.79^{c}	16.67±0.60^{c}
C	56.36±3.72^{a}	37.49±3.10^{b}	6.92±0.58^{c}	0.00±0.00^{d}
GCG	8.78±0.22^{a}	2.42±1.69^{b}	0.00±0.00^{c}	0.00±0.00^{c}
CG	6.58±0.22^{a}	5.22±0.48^{b}	4.35±0.13^{c}	2.89±0.14^{d}

Data are presented as the mean ± SD (n = 3). epigallocatechin (EGC), epicatechin (EC), epigallocatechin-3-gallate (EGCG), epicatechin-3-gallate (ECG),gallocatechin (GC), catechin (C), gallocatechin-3-gallate (GCG),and catechin gallate (CG). Values with different letters (a–d) differ from each other significantly ($p < 0.05$).

Table S2: The primer sequences of RT-PCR

Primer	Forward	Reverse
β-actin	TGTTACCAACTGGGACGACA	CTGGGTCATCTTTTCACGGT
Cckar	GATGCCAGCCAGAAGAAATC	ACAGCCATCGCTATCCTCAT
Drd2	ACTACCTGATCGTCAGCCTCG	ATGTCACAGTGAATCCTGCTG
Drd3	TGGGGCAGAAAACTCCACTG	TACCAGACCGTTGCCAAAGAT
Gabre	ACTGCGCCCTGGCATTGGAG	AGGCCCGAGGCTGTTGACAA
Mc2r	CACACCAATGACACCGCAAG	CACAGCCAGGAGGACAATCA
Oprk1	ATCACCGCTGTCTACTCTGTGG	GTGGTAGTAACCAAAGCATCTGC
Sstr5	TGGCGTCCTCCCTTCTTC	TGTTGTCACCGCCTCCA
Trh	CAGAACGTCGATTCTTGAGGAAAG	TCGGCTTCAACGTCTTCCTCCT
Chrnb3	ACGTGTGGCTGAAGCAGGAAT	CAGAGCGACTCCGATGGAAC
Fpr1	CCTTGGCTTTCTTCAACAGC	GCCCGTTCTTTACATTGCAT
Gal	ACCAGGAAGTGTTGATGTGCC	TCTAGGTCTTCTGAGGAGGTGG
Ghrh	GGTGCTCTTTGTGATCCTCATC	GTTTCCTGTAGTTGGTGGTGAAG

Supporting Figures

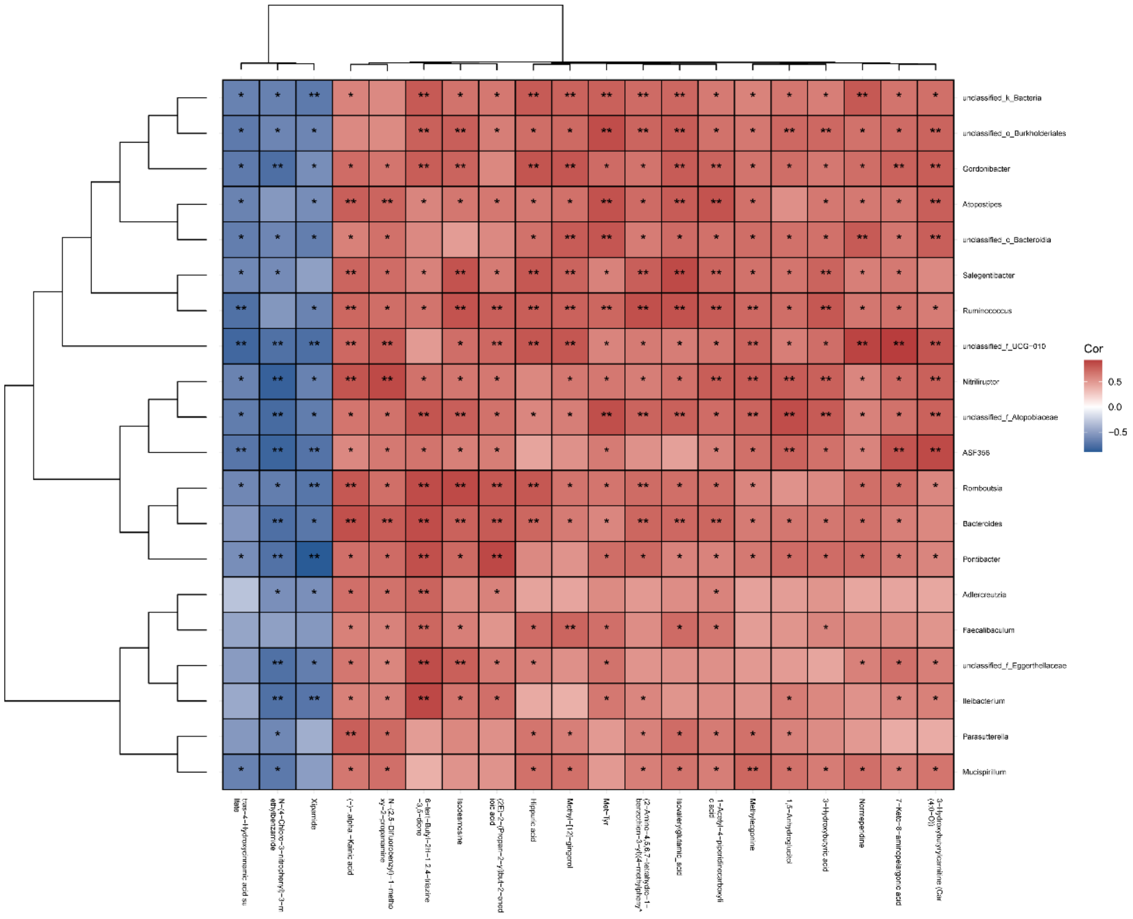

Figure S1. Hierarchical clustering heatmap of correlations between gut microbiota and serum metabolites. Asterisks indicate statistical significance: $p < 0.05$ (*) and $p < 0.01$ (**).
